# ASSOCIATION BETWEEN FAMILY TYPOLOGY AND COGNITIVE FUNCTION AMONG OLDER ADULTS IN THE US? FINDINGS FROM THE PINE STUDY

**DOI:** 10.1093/geroni/igz038.1384

**Published:** 2019-11-08

**Authors:** Mengting Li, Man Guo, Meredith Stensland, XinQi Dong

**Affiliations:** 1 Rutgers, The State University of New Jersey, New Brunswick, New Jersey, United States; 2 University of Iowa, Iowa, United States; 3 The University of Texas at Austin, Austin, Texas, United States; 4 Rutgers Institute for Health, Health Care Policy and Aging Research, New Brunswick, United States

## Abstract

A broad literature has explored racial and ethnic disadvantages in cognitive aging. Migration and acculturation created additional challenges on cognitive aging of minority older immigrants. Asian Americans are the fastest growing minority group in the United States. Chinese Americans constitute the largest segment of Asian Americans. Family is a core social value in Chinese culture. Less is known regarding the impact of family relationship on cognitive function for US Chinese older immigrants. Data were derived from the Population Study of Chinese Elderly (PINE), a community-engaged, population-based epidemiological study of 3,157 US Chinese older adults aged 60 and above in the greater Chicago area from 2011-2013. A typology approach is a useful tool to operationalize multifaceted family relationships. Our prior study used Latent Class Analysis to cluster family typologies, evaluating structural, associational, affectual, functional and normative aspects of family relationship. Cognitive function was evaluated by global cognition, episodic memory, executive function, working memory, and Chinese Mini-Mental State Examination (C-MMSE). Linear regression and quantile regression were used. The findings showed detached and commanding conflicted typologies were associated with lower global cognitive function compared with unobligated ambivalent typology. Wish respect to cognitive domains, detached, commanding conflicted, and tight-knit typologies were associated with lower episodic memory, working memory, and C-MMSE than unobligated ambivalent typology, respectively. Commanding conflicted typology, featured by high intergenerational conflicts, was associated with lowest cognitive function among all typologies. Health care professionals and social service providers should focus on older adults with commanding conflicted typology and prevent them from cognitive impairment.

